# Pathological Mechanistic Studies of Osimertinib Resistance in Non-Small-Cell Lung Cancer Cells Using an Integrative Metabolomics-Proteomics Analysis

**DOI:** 10.1155/2020/6249829

**Published:** 2020-03-17

**Authors:** Qing Ma, Jing Wang, Yaoyao Ren, Fanlu Meng, Lili Zeng

**Affiliations:** Tianjin key Laboratory of Lung cancer Metastasis and Tumor Microenvironment, Tianjin 300052, China

## Abstract

**Background:**

Osimertinib is the first-line therapeutic option for the T790M-mutant non-small-cell lung cancer and the acquired resistance obstructs its application. It is an urgent challenge to identify the potential mechanisms of osimertinib resistance for uncovering some novel therapeutic approaches.

**Methods:**

In the current study, the cell metabolomics based on ultra-high-performance liquid chromatography coupled with linear ion trap-Orbitrap mass spectrometry and the qualitative and tandem mass tags quantitative proteomics were performed.

**Results:**

54 differential metabolites and 195 differentially expressed proteins were, respectively, identified. The amino acids metabolisms were significantly altered. HIF-1 signaling pathway modulating P-glycoproteins expression, PI3K-Akt pathway regulating survivin expression, and oxidative phosphorylation were upregulated, while arginine and proline metabolism regulating NO production and glycolysis/gluconeogenesis were downregulated during osimertinib resistance.

**Conclusion:**

The regulation of HIF-1 and PI3K-Akt signaling pathway, energy supply process, and amino acids metabolism are the promising therapeutic tactics for osimertinib resistance.

## 1. Introduction

Lung cancer, especially non-small-cell lung cancer (NSCLC) which accounts for more than 80% of lung cancer cases [1], has become one of the most common and lethal cancers with 16% morbidity and 18.4% mortality among all kinds of malignant cancers [2]. Among the NSCLC patients, the percentage of epidermal growth factor receptor- (EGFR-) mutant cases is approximately 15% in Western countries and 40% in Asian countries [3]. For the EGFR-mutant NSCLC patients, EGFR-tyrosine kinase inhibitors (EGFR-TKIs), which can effectively bind to the ATP-binding pocket of the EGFR-tyrosine kinase domain to block downstream signaling pathway, have become the preferred targeted therapy [4–6]. During the first six months of administration, EGFR-mutant NSCLC patients are sensitive to first- and second-generation EGFR-TKIs, but then, the disease progresses in more than 50% of patients for the *p*.Thr790Met point mutating (T790M) in the gene encoding EGFR [7, 8]. For the remedy of T790M-positive NSCLC patients, the third-generation EGFR-TKIs, like osimertinib (AZD9291), were approved by the Food and Drug Administration in 2015 [9].

Osimertinib, as an irreversible EGFR-TKI, does influence the treatment strategy for T790M-mutant NSCLC patients with approximately 60% objective response rate and 9 months' duration of progression-free survival [9–11]. However, acquired resistance to osimertinib emerges one after another from 2015. The acquired resistance can be sorted into two kinds of alterations, one being EGFR alteration such as C797S mutation [12] and the other being alterations activating downstream signaling, bypassing EGFR, to maintain the oncogenic effect of EGFR, or starting parallel signaling pathway to promote cancer cells metastasis [13, 14]. Although some mechanisms of the acquired resistance have been identified, there are still many concealed resistance mechanisms which become the major obstacle for effective EGFR targeted therapy.

Systems biology has become a pivotal tool which integrates biological information from genes, proteins, and metabolites to discover the potential mechanisms of physiological and pathological states of organisms, tissues, or cells [15, 16]. In the current study, the metabolomics combined with proteomics analysis was applied to uncover the biological changes of non-small-cell lung cancer H1975 cells translating into osimertinib-resistant H1975 cells. This research will help to discover some novel osimertinib resistance mechanisms and provide some enlightenments on overcoming osimertinib resistance.

## 2. Materials and Methods

### 2.1. Materials

MTS cell proliferation colorimetric assay kit was purchased from Abcam (Cambridge, UK). Osimertinib was bought from Selleck Chemicals (TX, USA). UPLC grade methanol, acetonitrile, and analytical grade formic acid were acquired from Merk (Darmstadt, Germany). Deionized water was purified by the Milli-Q system (Bedford, MA, USA). Urea, trichloroacetic acid, iodoacetamide, and tris(s-carboxyethyl)-phosphine (TCEP) were purchased from Sigma-Aldrich (St. Louis, USA). Trypsin was obtained from Promega (WI, USA). Annexin V-FITC apoptosis detection kit was obtained from BD Biosciences (NJ, USA). Antibodies against pyruvate kinase PKM (PKM), integrin beta 3 (ITGB3), phosphoenolpyruvate carboxykinase (PCK2), and *β*-actin were obtained from Abcam (Cambridge, UK). Antibodies against ATP synthase protein (ATP8), plasminogen activator inhibitor 1 (SERPINE1), and glycine amidinotransferase (GATM) were bought from Cell Signaling Technology (MA, USA). The HRP-conjugated secondary antibody was acquired from Bioss, Inc. (Beijing, China). All reagents for cell cultures were bought from Gibco BRL Life Technologies (MD, USA) and all chemicals were analytical grade.

### 2.2. Cell Culture

Human non-small-cell lung adenocarcinoma cell lines (H1975) were purchased from American Type Culture Collection (ATCC, Rockville, MD, USA) and cultured in RPMI 1640 medium supplemented with 100 U/mL penicillin, 0.1 mg/mL streptomycin and 10% (v/v) foetal bovine serum, in a 37°C humidified incubator with 5% CO_2_. The H1975 cells were fed every day and were subcultured when they reached almost 80% confluency.

### 2.3. Establishment of Osimertinib-Resistant Cell Lines

The H1975 cells were plated in 96-well plate at a density of 1 × 10^4^ cells/well with complete medium and cultured overnight. To establish osimertinib-resistant (OR) cells, the cells were then incubated in medium containing 5 × 10^−8^ mol/L osimertinib for almost 60 h. After that, the cells were again cultured in fresh complete medium and the exponential growth cells were selected as potential OR cells. The potential OR cells experienced several cycles of being incubated in medium containing osimertinib and selected in exponential growth stage. During the cycles, the concentration of osimertinib increased from 5 × 10^−8^ mol/L to 5 × 10^−6^ mol/L. After 6 months of incubation in medium containing 5 × 10^−6^ mol/L osimertinib, the cells became OR cells. The OR cells were also fed every day and were subcultured when they reached almost 80% confluency.

### 2.4. Cell Viability Assay

3-(4,5-dimethylthiazol-2-yl)-5-(3-carboxymethoxyphenyl)-2-(4-sulfophenyl)-2H-tetrazolium (MTS) assay was conducted to investigate the cell viability of H1975 cells and OR cells under the treatment of osimertinib. The H1975 and OR cells were, respectively, plated in 96-well plate at a density of 1 × 10^5^ cells/well with complete medium for 24 h. Then, the H1975 and OR cells were both incubated in medium containing 0, 10^−10^, 10^−9^, 10^−8^, 10^−7^, 10^−6^, and 10^−5^ mol/L osimertinib for 72 h. After 72 h incubation, the H1975 and OR cells were cultured in MTS reagent for 4 h. Then, the absorbance was measured at optical density (OD) 490 nm by a microplate reader (Bio-RAD, CA, USA). The OD values of H1975 and OR cells untreated with osimertinib were considered as OD (untreated) and the cell viability was represented as cell growth (%) = OD (treated)/OD (untreated).

### 2.5. Cell Apoptotic Assay

The cells were cultured in a 6-well plate with the density of 2 × 10^5^ cells/well, by the complete medium for 24 h, and then stimulated by 10^−5^ mol/L osimertinib for 8 h. The cells were collected, washed by ice-cold PBS for three times, and then resuspended by 1x binding buffer. 100 *μ*L cell suspension was stained by 1/1000 (v/v) Annexin V-FITC for 20 min at room temperature, in a dark place. Next, the cells were washed by ice-cold PBS and stained with deliquated binding buffer containing 2 *μ*g/mL propidine iodide (PI). The apoptosis evaluation was conducted by flow cytometer (Beckman Coulter, CA, USA). Data were analyzed with FlowJo software (Tree Star, OR, USA).

### 2.6. Cell Cycle Assay

To assess the cell cycle arrest, H1975 and OR cells were cultured in 6-well plates in complete medium for 24 h and then stimulated by 10^−5^ mol/L osimertinib for 8 h. The cells were washed by PBS and fixed for 15 min at 4°C. The fixed cells were washed and stained by 50 *μ*g/mL PI in the presence of 100 *μ*g/mL RNase. The cells were then analyzed by flow cytometer (Beckman Coulter, CA, USA) and cell cycle profile was analyzed with ModFit software.

### 2.7. Sample Preparation and LC-MS Conditions for Metabolomics Analysis

A 100 *μ*L cell pellet was suspended in 500 *μ*L methanol precooled in −80°C. The cell suspension was vortexed for 30 s and then snap-freezed in liquid nitrogen. The quenched cells were thawed at room temperature, vortexed for 30 s, and then centrifugated at 800 ×g for 1 min. The cell supernatant was stored in a tube on dry ice. The freezing-thawing circle was repeated for the unquenched cells. All cell supernatants were collected in one tube and centrifugated at 15000 ×g for 1 min. The supernatant was blow-dried by vacuum concentration at 30°C and then dissolved by 300 *μ*L 2-chlorobenzalanine methanol aqueous solution (1 : 1, 4°C) for LC-MS analysis.

A Waters ACQUITY ultra-performance liquid chromatography (UPLC) system (Waters Corporation, Milford, USA) coupled with a linear ion trap (LTQ)-Orbitrap XL Mass Spectrometer from Thermo (Thermo Fisher Scientific, Inc.) was used. A Waters ACQUITY HSS T3 column (2.1 mm × 150 mm, 1.8 *μ*m) was applied for metabolites separation. The flow rate was 0.25 mL/min and the column was maintained at 40°C. A 4 *μ*L aliquot of each sample was injected into UPLC system. The optimal mobile phase consisted of a linear gradient system of (*A*) 0.1% formic acid in water and (*B*) 0.1% formic acid in acetonitrile: 0–1.0 min, *B* 0–2%; 1–9.5 min, *B* 2–50%; 9.5–14 min, *B* 50%–98%; 14–15 min, *B* 98%; 15–15.5 min, *B* 98–2%; 15.5–17 min, 2%. The mass spectra were acquired in both positive (ESI^+^) and negative (ESI^−^) ionization modes and the optimal conditions of analysis were set as follows: the spray voltage was 4.8 kV for positive mode and 4.5 kV for negative mode. Shear gas and auxiliary gas was set at 45 and 15 arbitrary units, respectively. The capillary temperature was 325°C and the capillary voltage was 3.5 kV for positive mode and 1.5 kV for negative mode. The voltage of tube was 50 V for positive mode and 50 V for negative mode. A full scan was acquired ranging from *m/z* 89 to 1000 Da at a resolution of 60,000. The collision energy was set at 30 eV and dynamic exclusion for previously fragmented precursor ions was applied with the repeat duration at 15 s.

### 2.8. Sample Repeatability and LC-MS System Stability

To ensure the accuracy of metabolomics results, the stability of LC-MS system and repeatability of metabolomics method were evaluated. For stability evaluation, the quality control (QC) sample, which was a pooled sample of each cell extraction, was injected into LC-MS system every six samples throughout the overall process and the QC samples clustered tightly in the principal component analysis (PCA) score plot (Supplementary [Supplementary-material supplementary-material-1]), which indicated that the stability of LC-MS system was excellent. For repeatability evaluation, the relative standard deviations (RSDs) of peak areas and retention times of six extracted ions in LC-MS spectra were analyzed. The RSDs of peak areas were less than 8.0% and RSDs of retention times were no more than 1.0%, which demonstrated that the repeatability of analysis method was good.

### 2.9. Metabolomics Data Analysis

The raw data were translated into mzXML format by Proteowizard package (v3.0.8786) and then disposed by R language (v3.3.2) for deconvolution, alignment, and data reduction. An unsupervised PCA for UV-scaled metabolite data matrix was conducted by SIMCA-P software (v13.0, Umetric, Umeå, Sweden) to assess the inherent characteristics of datasets from H1975 and OR groups. To discriminate groups and identify content differential variates between H1975 and OR cells, the supervised orthogonal projections to latent structures discriminant analysis (OPLS-DA) was performed and *p* value of the cross-validated analysis of variance (CV-ANOVA) was applied to assess its reliability.

To uncover the potential differential variates, an integration of S-plot and variable influence in the projection (VIP) plot of OPLS-DA analysis was performed. The potential metabolites were identified by aligning the exact molecular mass of differential variates with data from HMDB, METLIN, and KEGG databases, with standards of mass deviation <5 ppm and high i-Fit value. The MS/MS fragments, isotopic distribution, and retention time were also analyzed for metabolite identification. To evaluate the discriminatory ability of differential metabolites, the area under ROC curve (AUC) was accumulated and the cutoff AUC value was 0.8. A heatmap of differential metabolites, clustered by Euclidean distance, was constructed and the metabolic pathway analysis was performed by MetPA.

### 2.10. Protein Samples Preparation

100 *μ*L cell pellet was dissolved in 300 *μ*L lysis buffer (8 mol/L urea, 1% protease inhibitor cocktail) and sonicated three times by a high intensity ultrasonic processor (Scientz, Ningbo, China). The protein solution was centrifugated at 15000 ×g at 4°C for 10 min and the protein concentration of supernatant was detected by bicinchoninic acid (BCA) kit according to the manufacturer's instruction.

The protein solution was reduced with 5 × 10^−3^ mol/L dithiothreitol at 56°C for 30 min, and then it was alkylated with 11 × 10^−3^ mol/L iodoacetamide for 15 min at room temperature in the dark. To adjust the urea concentration to be less than 2 mol/L, the protein solution was diluted by 0.1 mol/L triethylbenzylammonium (TEAB). At last, the trypsin was added into protein solution with the ratio of 1 : 50 (w/w) for digestion overnight at 37°C. The protein samples were desalted by 5% formic acid and then every sample was labeled by 6-plex TMT kit as the manufacturer's instructions (Applied Biosystems, CA, USA).

### 2.11. LC-MS Conditions for Proteomics Analysis

The labeled peptides were dissolved in 10^−2^ mol/L ammonium acetate in water (pH = 10.0) (solvent *A*) and then loaded onto a Waters BEH C18 column (2.1 mm × 150 mm, 1.7 *μ*m). The flow rate was 0.25 mL/min and a 50 *μ*L aliquot of each sample was injected into UPLC system. The optimal mobile phase consisted of a linear gradient system of (*B*) 10^−2^ mol/L ammonium acetate in 90% acetonitrile (pH = 10.0): 0–35.0 min, *B* 0–45%; 35.0–37.0 min, *B* 45–80%; 37.0–40.0 min, *B* 80%; 40.0–42.0 min, *B* 80%-0; 42.0–45.0 min, *B* 0. Full-scan MS spectra (from *m*/*z* 400–1500) were gathered by the Orbitrap analyzer with a resolution of 60,000 at *m*/*z* 200. The automatic gain control was set at 3 × 10^6^. The 15 most intense ions were subjected to collisional induced dissociation with a normalized energy of 32%. The optimal mass spectrometry conditions were as follows: spray voltage was 2.2 kV. Capillary temperature was set at 320°C and normalized collision energy using wide-band activation mode 32% for MS2 was used. The ion selection threshold was minimum ion intensity of 50,000 and activation time was 80 ms.

### 2.12. Data Analysis of Proteome Information

The MS/MS spectra were filtered by Proteome Discoverer software (v2.1 thermo fisher scientific, Inc.) and then were searched against Uniprot-Swissprot database (Homo sapiens) by Proteome Discoverer sequent. The mass tolerance for precursor ions was 10 ppm and 50 mmu in MS/MS search. Carbamidomethyl on Cys was specified as fixed modification and oxidation on Met was specified as variable modifications. False-positive rate (FDR) by percolator algorithm was adjusted to <1%.

Gene ontology (GO) annotation proteome was derived from the Uniprot-GOA database (http://www.http://www.ebi.ac.uk/GOA/) and the Kyoto Encyclopedia of Genes and Genomes (KEGG) database was used to annotate protein pathways. The annotated KEGG pathways combined with differential metabolites that participated in the pathways was comprehensively analyzed.

### 2.13. Western Blot

The protein extraction procedures were similar to Section 2.10. The equivalent protein samples were subjected to 12% SDS-PAGE followed by western blot analysis. The PVDF membranes were blocked by 5% skim milk in TBST for 1 h at room temperature and then were incubated overnight at 4°C by the primary antibodies: anti-human PKM (1 : 1000), ATP8 (1 : 1000), ITGB3 (1 : 2000), GATM (1 : 1000), SERPINE1 (1 : 2000), PCK2 (1 : 1000), and *β*-Actin (1 : 1000). After primary antibodies incubation, the PVDF membranes were incubated with HRP-conjugated secondary antibodies for 1 h at room temperature. The proteins were stained with Immobilon™ Western Chemiluminescent HRP Substrate detection reagent (Millipore, MA, USA) and captured using Image Lab™ software (Bio-Rad, VA, USA). To quantify the relative intensity of proteins, the ratios of target proteins to *β*-actin signals were calculated by ImageJ software.

### 2.14. Statistical Analysis

All data were presented as the mean ± standard deviation (SD). The statistical analysis was performed by SPSS v.18.0 statistical analysis software (SPSS Inc., Chicago, USA). Statistical comparisons between H1975 and OR groups were conducted with an unpaired, two-tailed Student's *t*-test and *p* values <0.05 were considered significant.

## 3. Results

### 3.1. OR Losing Sensitivity to Osimertinib

**Figure 1:**
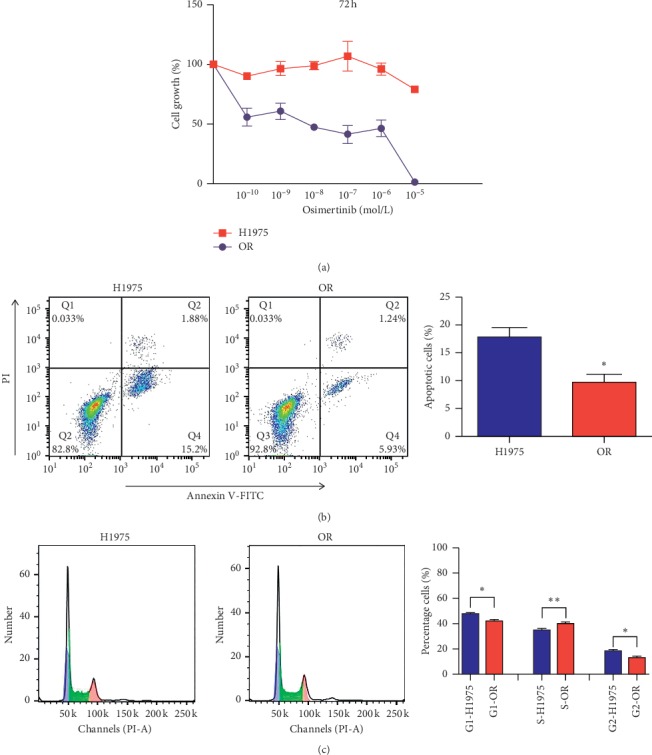
OR cells losing sensitivity to osimertinib. (a) The in vitro cell viability assay of OR and H1975 cells after incubation with 10^−5^-10^−9^ mol/L osimertinib for 72 h detected by MTS kit (*n* = 6). (b) The cell apoptotic assay of H1975 and OR cells after incubation with 10^−5^ mol/L osimertinib for 8 h (*n* = 3). (c) The cell cycle arrest assay of H1975 and OR cells after incubation with 10^−5^ mol/L osimertinib for 8 h (*n* = 3). Each bar represents the mean ± S.D. ^*∗*^*p* < 0.05, ^*∗∗*^*p* < 0.01, and ^*∗∗∗*^*p* < 0.001 compared to the H1975 group.

To investigate the sensitivity of H1975 cells and OR cells to osimertinib, the MTS assay was performed. The results demonstrated that H1975 cells were sensitive to osimertinib treatment and the cell growth (%) was decreased by osimertinib administration, with a concentration-dependent manner (Figure 1(a)). The cell growth (%) almost got close to 0 by 10^−5^ mol/L osimertinib administration. However, the osimertinib treatment barely interfered with the growth of OR cells, and the cell growth (%) was slightly decreased in 10^−5^ mol/L osimertinib (Figure 1(a)). These results indicated that OR cells have lost sensitivity to osimertinib.

The early apoptotic cells were stained only by Annexin V and located in the Q4 area in Figure 1(b), while the late apoptotic cells were stained by Annexin and PI and located in the Q2 area in Figure 1(b). The cell apoptosis evaluation included both the early apoptotic cells and the late apoptotic cells. After stimulation by 10^−5^ mol/L osimertinib for 8 h, the percentage of apoptotic H1975 cells was significantly higher than that of apoptotic OR cells (Figure 1(b)).

Compared with H1975 cells, the percentage of OR cells in G1 and G2 phase was significantly decreased, while the percentage of OR cells in S phase was increased, after stimulation by 10^−5^ mol/L osimertinib for 8 h (Figure 1(c)).

### 3.2. Classification of the Cell Metabolic Profiles and Differential Metabolites Identification

**Figure 2:**
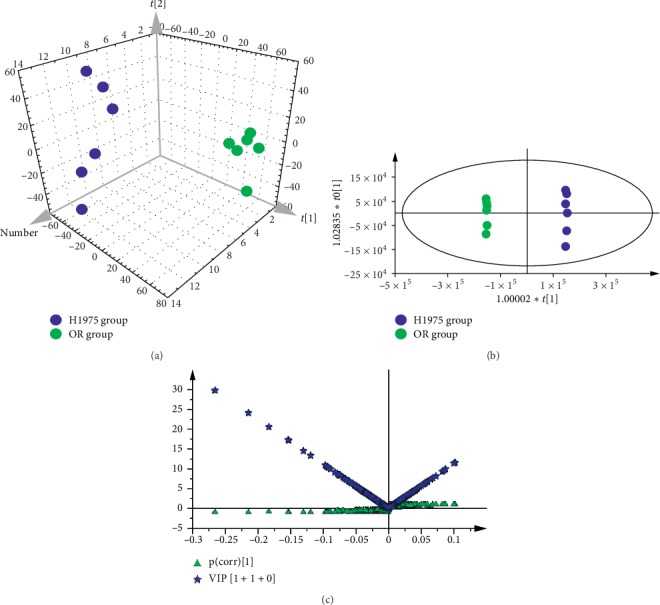
Efficacy classification models of the whole metabolism between H1975 and OR cells, in positive ion scan mode. (a) PCA 3D score plot of the H1975 and OR groups in positive ion scan mode (*n* = 6). (b) OPLS-DA score plot of the H1975 and OR groups in positive ion scan mode (*n* = 6). (c) A combination plot of loading S-plot and VIP-values for identifying differential metabolites.

**Figure 3:**
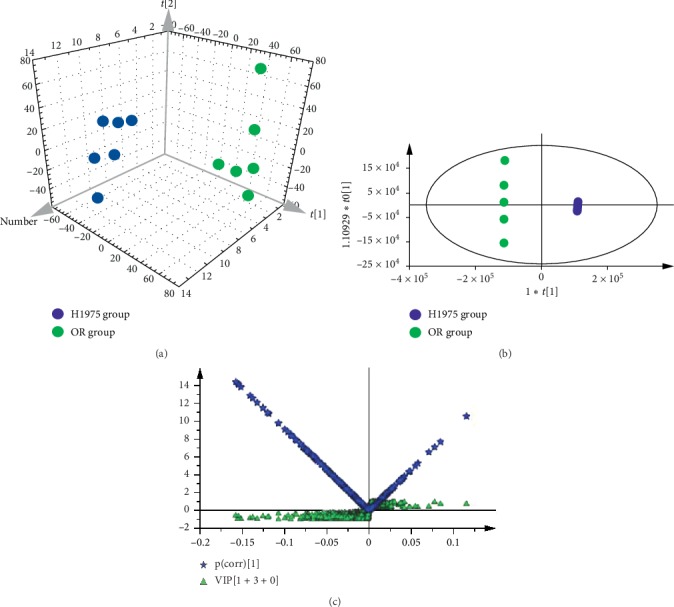
Efficacy classification models of the whole metabolism between H1975 and OR cells, in negative ion scan mode. (a) PCA 3D score plot of the H1975 and OR groups in negative ion scan mode (*n* = 6). (b) OPLS-DA score plot of the H1975 and OR groups in negative ion scan mode (*n* = 6). (c) A combination plot of loading S-plot and VIP- values for identifying differential metabolites.

**Figure 4:**
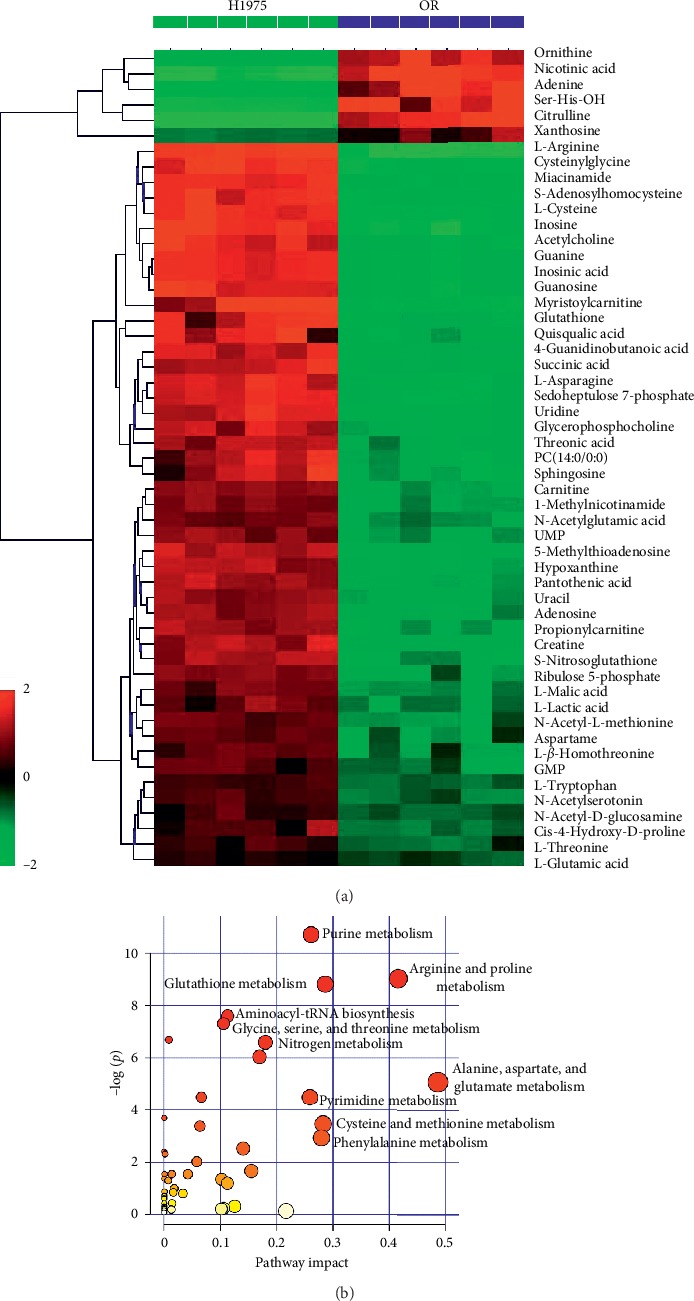
Differential metabolites and their corresponding pathways. (a) Heatmap of the intensities of differential metabolites in H1975 and OR cells (*n* = 3). The degree of change is marked with different colors: red indicates upregulation, and green represents downregulation. Each row represents an individual sample and each column represents a metabolite. (b) Metabolism pathways participating in osimertinib resistance and the map was generated by MetPA.

To distinguish the classes and to assess the global metabolism variations, the unsupervised PCA was performed in both positive and negative spectra. The PCA 3D score plot of positive spectra indicated that there was a clear classification of observations of H1975 cells and OR cells (Figure 2(a)). Similar to the PCA of positive spectra, the observations of H1975 cells and OR cells were clearly divided into two parts in the score plot, and the observations of OR cells were located in the left side and the observations of H1975 cells in right side, in the PCA of negative spectra (Figure 3(a)). To further distinguish the H1975 cells and OR cells and to identify differential variants, a supervised OPLS-DA was conducted. In Figure 2(b), a remarkable separation of positive data in H1975 and OR groups was observed in the OPLS-DA score plot (*R*^2^*X* = 0.423, *R*^2^*Y* = 1, *Q*^2^ = 0.95) and the CV-ANOVA *p* value was 1.221*E*^−4^, which suggested that this OPLS-DA model was non-overfitting. In Figure 3(b), the OPLS-DA score plot (*R*^2^*X* = 0.565, *R*^2^*Y* = 1, *Q*^2^ = 0.911, CV-ANOVA = 0.01635) of negative data showed that observations of OR group clustered in the right quadrant and observations of H1975 group distributed in the left quadrant. The differential variants were screened and identified from the loading S- and VIP-value plots of the OPLS-DA, of both positive and negative modes (Figures 2(c) and 3(c)). The corresponding metabolites of differential variants were validated by the univariate ROC curve analysis with the threshold 0.8. Fifty-four differential metabolites were identified and the heatmap of the differential metabolites were shown in Figure 4(a).

### 3.3. Pathway Analysis Based on Differential Metabolites

To uncover the most relevant biological pathways of osimertinib resistance, the ingenuity network analysis by MetPA was performed. The enrichment and topology analysis demonstrated that osimertinib resistance is related to amino acid metabolisms, especially arginine and proline metabolism, purine metabolism, and alanine, aspartate, and glutamate metabolism (Figure 4(b)).

### 3.4. Differentially Expressed Proteins Identification and GO Functional Analysis

In the current study, we performed a TMT-labeled quantitative proteomics approach to investigate the potential mechanism of osimertinib resistance. The raw data were searched against Uniprot-Swissprot database (Homo sapiens) and approximately 5000 proteins per sample were identified with the FDR less than 1%.

**Figure 5:**
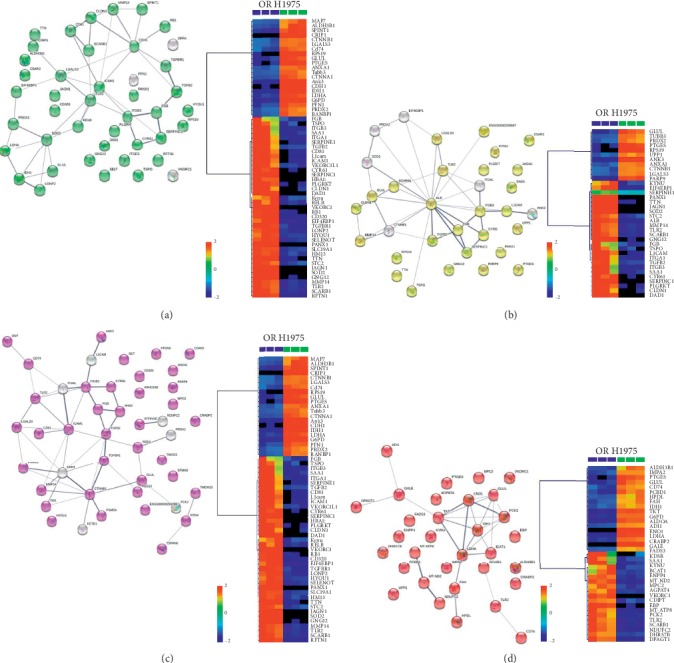
Five significantly altered GO pathways, after osimertinib resistance in H1975 cells. (a) The protein-protein interaction of differentially expressed proteins in response to chemical and the heatmap of intensities of those differentially expressed proteins (*n* = 3). (b) The protein-protein interaction of differentially expressed proteins in response to external stimulus and the heatmap of intensities of those differentially expressed proteins (*n* = 3). (c) The protein-protein interaction of differentially expressed proteins in regulation of cell communication and the heatmap of intensities of those differentially expressed proteins (*n* = 3). (d) The protein-protein interaction of differentially expressed proteins in small molecule metabolic process and the heatmap of intensities of those differentially expressed proteins (*n* = 3). The degree of change is marked with different colors: red indicates upregulation, and blue represents downregulation. Each row represents an individual sample and each column represents a metabolite.

**Figure 6:**
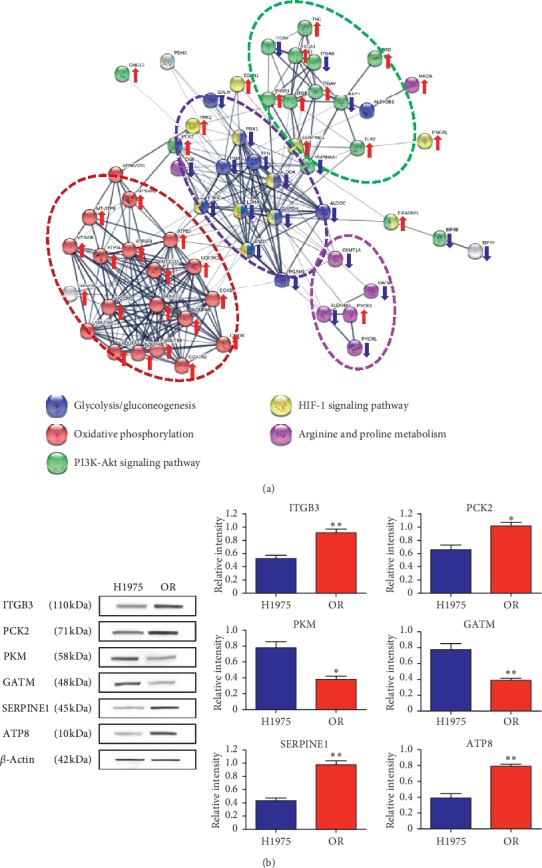
(a) The protein-protein interaction of key differentially expressed proteins and the vital KEGG pathways that those proteins are enriched in. Each KEGG pathway was marked by one color. The expression variation of proteins was labeled by arrows. (b) The protein expression of some key differentially expressed proteins analyzed by Western blots. (*n* = 3). Each bar represents the mean ± S.D. ^*∗*^*p* < 0.05, ^*∗∗*^*p* < 0.01, and ^*∗∗∗*^*p* < 0.001 compared to the H1975 group.

The differentially expressed proteins were further filtered according to the fold-change with more than two criteria and *p* < 0.05. Based on the differentially expressed proteins, gene ontology (GO) analysis was conducted. The results indicated that 195 differentially expressed proteins were identified (Supplementary [Supplementary-material supplementary-material-1]) and these proteins were mainly enriched in four GO pathways, including response to chemical (61 proteins, FDR = 1.36*e*^−5^), response to external stimulus (39 proteins, FDR = 2.25*e*^−5^), regulation of cell communication (52 proteins, FDR = 1.6*e*^−3^), and small molecule metabolic process (38 proteins, FDR = 1.27*e*^−6^) (Figure 5). From the results of GO analysis, osimertinib resistance was mainly related to cell response to chemical or external stimulus and small molecule metabolic process such as amino acid metabolisms. Beside the GO analysis, KEGG analysis indicated that osimertinib resistance was closely associated with pathways as glycolysis/gluconeogenesis, oxidative phosphorylation, PI3K-Akt signaling pathway, HIF-1 signaling pathway, and arginine and proline metabolism. During those pathways, HIF-1 signaling pathway, PI3K-Akt signaling pathway, and oxidative phosphorylation were upregulated, while arginine and proline metabolism and glycolysis/gluconeogenesis were downregulated (Figure 6(a)). The corresponding protein variation trends were labeled in Figure 6(a) by arrows and shown in Supplementary [Supplementary-material supplementary-material-1].

### 3.5. Validation of Proteins Expression by Western Blots

In order to validate the signaling molecules' variation identified in the above omics, the expression of ITGB3, PCK2, PKM, GATM, SERPRINE1, and ATP8 was analyzed by western blots. The results indicated that the expression of ITGB3, PCK2, SERPRINE1, and ATP8 was obviously increased in OR cells, while the expression of PKM and GATM in OR cells was significantly decreased (Figure 6(b)). This result was similar to the variation tendency of signaling molecules observed in the above omics analysis, which indicated that the results observed in metabolomics-proteomics analysis were reliable.

## 4. Discussion

Activation of downstream, parallel, or auxiliary signaling pathway of EGFR, such as MET amplification and activation of RAS signaling, plays pivotal roles in the osimertinib resistance [17]. Survivin, a prosurvival factor, promoted the resistance against EGFR-TKIs [18, 19] and decreased survivin expression by inhibiting PI3K-Akt signaling pathway and enhanced the chemosensitivity of cancer stem cells to EGFR-TLIs [19]. Moreover, blocking of mTOR activity reinforced the sensitivity to EGFR-TKIs in glioma cells, while mTOR is also a downstream signaling molecule of PI3K-PTEN-Akt pathway [20]. In the current results, ten differentially expressed proteins, participating in PI3K-AKT pathway, were upregulated, which indicated that the PI3K-AKT signaling pathway was upregulated in OR cells, compared with H1975 cells. Furthermore, the content of AMP was significantly decreased in OR cells, which might be partly due to participating in mTOR signaling pathway to promote proteins synthesis.

Hypoxia-inducible factors (HIFs), a kind of transcription factors activated under the hypoxic environment to maintain cell activity, exert an important effect on tumor's drug resistance [21]. HIF-1 can induce the expression of P-glycoprotein through binding to the HIF-1 binding site in MDR1 promoters, and P-glycoprotein is the multidrug efflux transporter in cells [22, 23]. Therefore, HIF-1 signaling pathway is closely associated with multidrug resistance. Some researches indicated that, under the hypoxic circumstance, the expression of HIF-1, P-glycoprotein in human pulmonary cancer was increased and drug resistance was also strengthened [24]. In our study, the expression of IFN-*γ* receptor and 4EBP1, upstream proteins of HIF-1, was statistically increased in OR cells, which suggested the upregulated HIF-1 signaling pathway and the promotion of HIF-1 expression. Therefore, the expression of TFRC associated with energy delivery was upregulated. However, compared with H1975 cells, the expression of ENO1 and GAPDH, related to anaerobic metabolism, was decreased in OR cells. Different from normal cells, the cancer cells always derive glucose from microenvironment and transport excess glucose mostly to glycolysis rather than oxidative phosphorylation, which provide almost 80% ATP to normal cells [25]. However, some reports supported that oxidative phosphorylation also played vital roles in the cancer progress [26, 27]. A group of slow-cycling cells isolated from different tumors exhibited characteristics of tumorigenic potential and multidrug resistance and those slow-cycling cells had a common metabolic process as the energy production depending on mitochondrial oxidative phosphorylation rather than glycolysis [28]. In our research, the glycolysis pathway was downregulated with 12 differentially expressed proteins being decreased, and the oxidative phosphorylation was significantly upregulated, which indicated that the metabolic mode of OR cells was exactly similar to those slow-cycling cells. Some researches also demonstrated that the combination of oligomycin (a complex V mitochondrial inhibitor) and tyrosine kinase inhibitors would produce an extinct effect of oncogenic signaling, which supported our results that OR cells had a restored oxidative phosphorylation [29]. Therefore, there was a reverse in the energy supply landscape when the cancer cells translated to osimertinib resistance cells.

Another crucial part of energy supply is amino acids metabolism. Amino acids are important participants and regulators of energy homeostasis, mitochondrial respiration, and superoxide production [30, 31]. In this research, the results of metabolomics indicated that amino acids metabolism obviously altered and the results of proteomics demonstrated that arginine and proline metabolism was downregulated in OR cells. Arginine is a major source for the synthesis of NO, proline, creatine, and many other metabolites and among them, NO exerts vital effects on the regulation of cancer progression [32, 33]. NO, at low levels, can promote cancer cells proliferation and inhibit apoptosis, while it suppresses cell proliferation of pheochromocytoma PC12 cells and facilitates cancer cells apoptosis at high concentrations [34, 35]. Thus, the decreased arginine content and downregulated arginine metabolism indicated the lower NO production in OR cells than that in H1975 cells, which suggested mighty proliferation and viability potential of OR cells.

## 5. Conclusion

Overall, in the study, a multiomics tactics by integrating metabolomics and proteomics was performed to identify the potential mechanism of osimertinib resistance of human non-small-cell lung adenocarcinoma cell. Our results demonstrated that 54 differential metabolites were identified in OR cells and those differential metabolites were mostly located in amino acids metabolism pathways. Besides, 195 differentially expressed proteins were identified in OR cells and they were enriched in GO pathways, including response to chemical, response to external stimulus, regulation of cell communication, and small molecule metabolic process. Osimertinib resistance was accompanied by the upregulated HIF-1 signaling pathway, PI3K-Akt signaling pathway and oxidative phosphorylation, and the downregulated glycolysis/gluconeogenesis and arginine and proline metabolism. Those alterations indicated increased survivin and P-glycoproteins, decreased anaerobic metabolism, and NO production in OR cells, compared with H1975 cells. Therefore, our results suggested that targeting survivin and P-glycoproteins production and energy supply process may be promising therapeutic approaches to prevent sensitive cancer cells from developing osimertinib resistance.

## Data Availability

All the data used to support the findings of this study are available from the corresponding author upon request.

## Supplementary Materials

Supplementary Materials In the supplementary materials, the QC samples are clustered tightly in the PCA score plot in both positive and negative modes, which indicated that the stability of LC-MS system was excellent. In the OPLS-DA assay, the differentially expressed proteins and the enriched KEGG pathways between H1975 cells and OR cells are shown in the tables.
