# Supplementary material for: Pathological Mechanistic Studies of Osimertinib Resistance in Non-Small-Cell Lung Cancer Cells Using an Integrative Metabolomics-Proteomics Analysis
Source: J Oncol. 2020 Mar 17;2020:6249829. doi: 10.1155/2020/6249829 (PMC7103047; doi:10.1155/2020/6249829)
Supplement: Supplementary Materials — In the supplementary materials, the QC samples are clustered tightly in the PCA score plot in both positive and negative modes, which indicated that the stability of LC-MS system was excellent. In the OPLS-DA assay, the differentially expressed proteins and the enriched KEGG pathways between H1975 cells and OR cells are shown in the tables. [file 6249829.f1.docx]

**Supplementary Materials**

**Pathological mechanistic studies of osimertinib resistance in non-small-cell lung cancer cells using an integrative metabolomics-proteomics analysis**

Qing Ma^1^*, Jing Wang^1^, Yaoyao Ren^1^, Fanlu Meng^1^, Liyan Gu^1^

*^1^Department of Medical Oncology, Tianjin Medical University General Hospital, Tianjin 300052, China.*

Running Head: Pathological mechanistic studies of osimertinib resistance by omics

Supplementary Figure 1

The PCA score plots of QC samples. (A) The PCA score plot of QC samples in positive mode. (B) The PCA score plot of QC samples in negative mode.


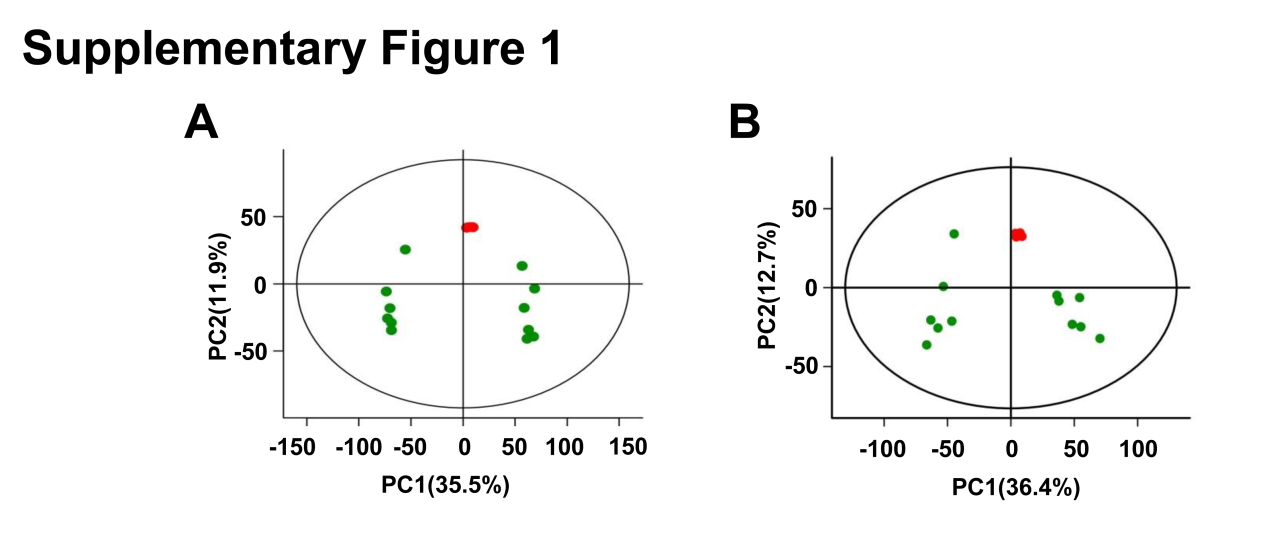


Table 1. The differential expressed proteins between H1975 cells and OR cells.

| Proteins | FDR | Log2(Fold change) | Proteins | FDR | Log2(Fold change) | Proteins | FDR | Log2(Fold change) |
| --- | --- | --- | --- | --- | --- | --- | --- | --- |
| CRIP1 | 0.000174 | -3.64933 | ARHGDIB | 0.000174 | -1.27459 | DBNL | 0.000293 | -1.09347 |
| EPCAM | 0.000141 | -2.79296 | ESRP1 | 0.000322 | -1.27386 | IMPA2 | 0.000399 | -1.08534 |
| TMSB4X | 8.95E-05 | -2.68209 | SPINT1 | 0.000174 | -1.26455 | ALDOA | 0.000174 | -1.07982 |
| CRABP2 | 0.00025 | -1.99354 | TKT | 0.000174 | -1.25819 | SERF2 | 0.001006 | -1.0707 |
| GALNT12 | 0.000174 | -1.94083 | PTMA | 0.000219 | -1.25803 | SAP30BP | 0.000174 | -1.07027 |
| ADI1 | 0.000174 | -1.89685 | SULT1A4 | 0.000174 | -1.25679 | ANXA1 | 0.000312 | -1.06818 |
| S100A4 | 0.000174 | -1.79856 | CLTA | 0.000265 | -1.25545 | RANBP1 | 0.000434 | -1.06207 |
| SERPINB5 | 0.002864 | -1.74879 | PCBD1 | 0.000474 | -1.23544 | CSTB | 0.001448 | -1.05727 |
| CDH1 | 0.000207 | -1.67958 | TMSB10 | 0.000402 | -1.22867 | C4A | 0.00097 | -1.0561 |
| RPS19 | 0.000446 | -1.67278 | LDHA | 0.000174 | -1.22407 | FAH | 0.000436 | -1.0516 |
| KRT19 | 0.000141 | -1.62603 | ITGB4 | 0.000146 | -1.21293 | TUBB3 | 0.000219 | -1.04935 |
| TXNDC17 | 0.010161 | -1.58435 | SERPINB1 | 0.000285 | -1.21173 | FSCN1 | 0.000367 | -1.04363 |
| ANK3 | 0.000125 | -1.57526 | ECHDC3 | 0.001482 | -1.19122 | HPDL | 0.000337 | -1.04168 |
| CKB | 0.000448 | -1.52912 | CAST | 0.000294 | -1.18774 | LGALS3 | 0.000375 | -1.03759 |
| PPIA | 0.000174 | -1.52189 | STMN3 | 0.000808 | -1.17874 | TAF15 | 0.000628 | -1.03347 |
| ENO1 | 0.00019 | -1.5112 | PFN1 | 0.000365 | -1.16689 | UPP1 | 0.000501 | -1.03 |
| JPT1 | 0.000174 | -1.50987 | AP1M2 | 0.000354 | -1.14958 | MAP7 | 0.000285 | -1.02784 |
| PAK6 | 0.000174 | -1.49806 | CKMT1A | 0.000987 | -1.14188 | CTNNB1 | 0.000678 | -1.02569 |
| PPL | 0.002402 | -1.43403 | WIPF2 | 0.000323 | -1.1409 | KCTD12 | 0.000354 | -1.02521 |
| RPL29 | 0.000354 | -1.41808 | LAD1 | 0.000396 | -1.1294 | PRDX2 | 0.000613 | -1.02498 |
| IDH1 | 0.000342 | -1.4054 | FHOD1 | 0.000482 | -1.1293 | PSMD9 | 0.000181 | -1.01886 |
| PTGES | 0.00081 | -1.40433 | ALDH3B1 | 0.000402 | -1.12384 | PROM2 | 0.01101 | -1.0099 |
| CAPS | 0.000285 | -1.36211 | CTNNA1 | 0.000246 | -1.11862 | C1QTNF6 | 0.000676 | -1.00315 |
| G6PD | 0.000328 | -1.35967 | DDT | 0.000501 | -1.11277 | GALE | 0.000174 | -1.00088 |
| PARP9 | 0.000181 | -1.35617 | PPHLN1 | 0.000626 | -1.10951 | DHRS7B | 0.000249 | 1.002284 |
| CGN | 2.95E-05 | -1.35014 | PEG10 | 0.000219 | -1.09871 | ATP6V0C | 0.000421 | 1.004057 |
| AKR1C3 | 0.000265 | -1.34203 | CD74 | 0.000932 | -1.09622 | TMEM205 | 0.000298 | 1.005683 |
| GLUL | 0.000848 | -1.31615 | HAUS5 | 0.000588 | -1.09538 | TGFB2 | 0.000896 | 1.012345 |
| CNBP | 0.000174 | -1.28201 | PKP3 | 0.000141 | -1.09437 | CDIPT | 0.000713 | 1.014108 |
| TMEM11 | 0.001474 | 1.018495 | MCAM | 0.001167 | 1.024865 | TPBG | 0.001149 | 1.016249 |
| KDSR | 0.00349 | 1.022539 | FGB | 0.011588 | 1.025289 | TENM3 | 0.000409 | 1.016999 |
| AHSG | 0.012757 | 1.027754 | ICOSLG | 0.002867 | 1.135971 | TOMM22 | 0.000185 | 1.360619 |
| QSOX2 | 0.000501 | 1.028014 | SCARB1 | 0.000392 | 1.140487 | CNN1 | 0.010683 | 1.374715 |
| BCAT1 | 0.001407 | 1.028616 | PFDN6 | 0.001149 | 1.141462 | ICAM1 | 0.001096 | 1.390523 |
| STC2 | 0.000589 | 1.040069 | BET1L | 0.004906 | 1.157367 | LSM2 | 0.000337 | 1.39788 |
| KYNU | 0.00262 | 1.046007 | TMEM33 | 0.005561 | 1.157952 | ALB | 0.00051 | 1.398577 |
| MPC2 | 0.000654 | 1.046786 | CLPTM1L | 0.000581 | 1.160538 | CD320 | 0.000467 | 1.406359 |
| PFDN5 | 0.001851 | 1.048263 | TPP1 | 0.003519 | 1.165786 | MPDU1 | 0.000146 | 1.408364 |
| HYOU1 | 0.004658 | 1.049674 | RTN3 | 0.001048 | 1.169478 | NMES1 | 0.001179 | 1.409169 |
| SLC37A4 | 0.000431 | 1.061554 | TSPAN6 | 0.00139 | 1.174607 | RB1 | 0.001679 | 1.414436 |
| MANBA | 0.00019 | 1.062415 | LONP2 | 0.000475 | 1.192655 | HSBP1 | 0.000365 | 1.420926 |
| AGPAT4 | 0.000353 | 1.066388 | TSPO | 0.008997 | 1.20398 | DPY30 | 0.005729 | 1.428678 |
| YIF1A | 0.00154 | 1.067487 | EIF4EBP1 | 0.000462 | 1.205629 | CLDN1 | 0.005126 | 1.446885 |
| SLC19A1 | 0.000174 | 1.070517 | MT-ATP8 | 0.001048 | 1.213639 | PLGRKT | 0.00201 | 1.469231 |
| JSRP1 | 0.002848 | 1.072211 | RELB | 0.00085 | 1.217878 | CD81 | 0.001112 | 1.482314 |
| HM13 | 0.000355 | 1.073701 | ITGA1 | 0.001776 | 1.222177 | ATP5MG | 0.000786 | 1.497852 |
| TM9SF3 | 0.001514 | 1.073979 | MAOA | 0.000505 | 1.226321 | SERPINE1 | 0.001974 | 1.502279 |
| TIMM8A | 0.000475 | 1.075237 | SLC7A5 | 0.000285 | 1.238521 | L1CAM | 0.000708 | 1.511079 |
| PI4KB | 0.003583 | 1.077001 | MT-ND2 | 0.000392 | 1.242565 | FADS3 | 0.032654 | 1.516932 |
| PCK2 | 0.005979 | 1.094841 | NDUFC2 | 0.000751 | 1.246233 | TTN | 0.000322 | 1.530478 |
| VKORC1L1 | 0.000968 | 1.09524 | ITGB3 | 0.003489 | 1.248527 | TIMM9 | 0.005654 | 1.553993 |
| SNRPE | 0.000392 | 1.09584 | PODXL | 0.000877 | 1.251173 | CYR61 | 0.000889 | 1.569165 |
| MANBAL | 0.002392 | 1.097332 | PALM2 | 0.00051 | 1.251435 | BANF1 | 0.000174 | 1.583502 |
| RTN4 | 0.000553 | 1.105045 | TGFBR1 | 0.001151 | 1.261135 | TOMM7 | 0.003809 | 1.59991 |
| MMP14 | 0.000399 | 1.11987 | SAA1 | 0.003669 | 1.262023 | TAGLN | 0.000808 | 1.601441 |
| SERPINB2 | 0.000986 | 1.123278 | RFTN1 | 0.000396 | 1.272722 | JAGN1 | 0.000207 | 1.603408 |
| SELENOT | 0.00019 | 1.124111 | ZFPL1 | 0.000447 | 1.302466 | VKORC1 | 0.000354 | 1.626355 |
| ENPP4 | 0.000482 | 1.132387 | DPAGT1 | 0.000482 | 1.306807 | PANX1 | 0.003721 | 1.633596 |
| CCDC88C | 0.000708 | 1.13263 | IGFBP7 | 0.000683 | 1.344007 | EBP | 0.00141 | 1.646644 |
| TLR2 | 0.000436 | 1.132676 | ATP5MPL | 0.000367 | 1.349059 | BRD9 | 0.000392 | 1.660466 |
| ASPHD1 | 0.000778 | 1.134175 | LSM5 | 0.000703 | 1.352561 | GNG12 | 0.000761 | 1.716817 |
| SF3B5 | 0.000392 | 1.358144 | SERPINC1 | 0.000932 | 1.761549 | SERPINC1 | 0.000932 | 1.761549 |
| C9 | 0.000613 | 2.287176 | TIMM8B | 0.037946 | 1.799862 | TIMM8B | 0.037946 | 1.799862 |
| ULK1 | 0.000416 | 2.297426 | ATP11A | 0.000474 | 1.847975 | ATP11A | 0.000474 | 1.847975 |
| SNRPF | 0.000207 | 2.543809 | HBA1 | 0.000753 | 2.642628 |  |  |  |

Fold change = content of differential expressed protein in OR cells/ content of differential expressed protein in H1975 cells

Table 2. Differential expressed proteins enriched in Key KEGG pathways.

| Proteins | Log2(Fold change) | Proteins | Log2(Fold change) | Proteins | Log2(Fold change) |
| --- | --- | --- | --- | --- | --- |
| Glycolysis / Gluconeogenesis | | | | | |
| PCK2 | 1.094841 | PDHX | 0.590061 | GAPDH | -0.71183 |
| PGK1 | -0.77963 | GALM | -0.78109 | ALDOC | -0.80366 |
| PKM | -0.85151 | ENO2 | -0.85159 | TPI1 | -0.91439 |
| PGAM1 | -0.93335 | LDHA | -1.22407 | ENO1 | -1.5112 |
| ALDOA | -1.07982 | ALDH3B1 | -1.12384 |  |  |
| Oxidative phosphorylation | | | | | |
| ATP5MG | 1.49785249 | COX5A | 0.703188957 | NDUFB5 | 0.859771137 |
| NMES1 | 1.409168527 | MT-ND5 | 0.701629667 | NDUFA5 | 0.858250363 |
| NDUFC2 | 1.246232687 | UQCRQ | 0.69665354 | NDUFB4 | 0.858151483 |
| MT-ATP8 | 1.21363877 | UQCRC2 | 0.659301867 | ATP5PF | 0.821974747 |
| ATP6V0C | 1.004057357 | COX6C | 0.64745939 | NDUFA3 | 0.79418793 |
| ATP6V0D1 | 0.998479817 | MT-CO2 | 0.59514949 | COX7A2 | 0.777884897 |
| NDUFB5 | 0.859771137 | ATP5PB | 0.713622033 | NDUFA9 | 0.722107243 |
| PI3K-Akt signaling pathway | | | | | |
| ITGB4 | -1.21293185 | TLR2 | 1.13267644 | EIF4B | -0.589806093 |
| RAC1 | -0.835202913 | EIF4EBP1 | 1.20562935 | BAD | 0.60579086 |
| ITGA6 | -0.806542307 | ITGA1 | 1.22217663 | THBS1 | 0.774873253 |
| EIF4H | -0.804382547 | ITGB3 | 1.248527317 | TNC | 0.885597193 |
| HSP90AA1 | -0.684859297 | GNG12 | 1.716816763 | ITGAV | 0.939998403 |
| EIF4B | -0.589806093 | PCK2 | 1.09484122 |  |  |
| HIF-1 signaling pathway | | | | | |
| ENO1 | -1.511202407 | EGLN1 | -0.641797803 | EIF4EBP1 | 1.20562935 |
| ENO2 | -0.851591847 | TFRC | 0.65261611 | SERPINE1 | 1.50227934 |
| GAPDH | -0.711828487 | IFNGR1 | 0.709749957 |  |  |
| Arginine and proline metabolism | | | | | |
| PYCR1 | 0.624588037 | PYCR3 | -0.93032359 | CKB | -1.529119317 |
| GATM | -0.823700683 | CKMT1A | -1.141876313 | MAOA | 1.226321213 |
| ALDH4A1 | 0.644812367 |  |  |  |  |

Fold change = content of differential expressed protein in OR cells/ content of differential expressed protein in H1975 cells
